# Vascular Behçet’s Disease: A Case of Arterial Occlusion and Successful Medical Management

**DOI:** 10.1155/carm/5576720

**Published:** 2026-02-12

**Authors:** Alhareth M. Amro, Leen M. Safadi, Huda Barqawi, Ammar Shaheen, Saed I. Atawnah

**Affiliations:** ^1^ Faculty of Medicine, Al-Quds University, Jerusalem, State of Palestine, alquds.edu; ^2^ Hebron University, Hebron, State of Palestine, hebron.edu; ^3^ Beit Jala Hospital, Bethlehem, State of Palestine; ^4^ Al-Quds University, Jerusalem, State of Palestine, alquds.edu; ^5^ Affiliated to Al-Ahli Hospital, Hebron, State of Palestine

**Keywords:** arterial occlusion, Behçet’s disease, case report, corticosteroids, immunosuppressive therapy, vascular inflammation

## Abstract

Behçet’s disease is a rare, chronic, multisystem vasculitis that can involve arteries and veins of all sizes, with vascular manifestations representing some of its most severe and potentially life‐threatening complications. Arterial involvement is uncommon but clinically significant, particularly in young patients. We report the case of a 26‐year‐old Palestinian male with a history of recurrent oral and genital ulcerations who presented with persistent left inguinal and proximal thigh pain accompanied by localized inflammatory signs. Laboratory investigations revealed elevated inflammatory markers and leukocytosis. Computed tomography angiography demonstrated complete occlusion of the left common femoral, external iliac, profunda femoris, and superficial femoral arteries, without evidence of distal ischemia or tissue compromise. Based on the combination of characteristic mucocutaneous manifestations and imaging findings, a diagnosis of vascular Behçet’s disease was established. The patient was treated conservatively with high‐dose systemic corticosteroids in combination with azathioprine, along with low‐dose aspirin. He showed rapid clinical improvement, with complete resolution of symptoms and stabilization of vascular lesions on follow‐up imaging. No surgical or endovascular intervention was required. This case highlights the importance of considering Behçet’s disease in young patients presenting with unexplained arterial occlusion and demonstrates that, in carefully selected cases without critical ischemia, timely immunosuppressive therapy may achieve favorable clinical and radiological outcomes while avoiding invasive interventions.

## 1. Introduction

Behçet’s disease (BD) is a chronic, multisystem inflammatory vasculitis that can involve arteries and veins of all sizes and types, with clinical expression influenced by gender, ethnicity, and geographic distribution [1]. It is an idiopathic multisystem inflammatory disorder characterized by a relapsing–remitting course with recurrent inflammatory flares affecting multiple organ systems [2]. Unlike other forms of vasculitis, BD is unique in its ability to affect both the arterial and venous circulations, with venous involvement occurring more frequently than arterial disease [3].

BD is more prevalent in regions along the historical Silk Road, including the Mediterranean basin, the Middle East, and East Asia, where it represents an important cause of inflammatory vascular disease in young adults [4]. The clinical spectrum of BD is broad and classically includes recurrent oral and genital ulcerations, ocular inflammation, and vascular involvement, while other systems may also be affected.

The International Study Group for Behçet’s Disease proposed classification criteria in 1990 based on recurrent oral ulceration in combination with additional clinical features, including genital ulceration, ocular involvement, characteristic skin lesions, or a positive pathergy test [5]. Although these criteria were developed primarily for research classification purposes and have since been revised, they remain widely used as a supportive framework in clinical practice when evaluating patients with suspected BD.

Beyond the original 1990 International Study Group (ISG) criteria, subsequent revisions have aimed to enhance diagnostic sensitivity, particularly in patients with atypical or vascular‐predominant disease. The International Criteria for Behçet’s Disease (ICBD), initially proposed in 2006 and revised in 2013, employ a weighted scoring system encompassing oral aphthosis, genital aphthosis, ocular involvement, skin lesions, neurological manifestations, and vascular involvement. These revised criteria have demonstrated improved sensitivity while preserving acceptable specificity across diverse clinical presentations. In the present case, the diagnosis of BD was supported by recurrent oral and genital ulcerations in conjunction with extensive arterial involvement, consistent with the ICBD classification framework and reinforcing the methodological robustness of the clinical assessment.

Vascular involvement is among the most severe manifestations of BD and is a major determinant of morbidity [6]. Venous disease predominates, with deep venous thrombosis of the lower extremities representing the most frequently affected site. Arterial involvement is less common, reported in a minority of patients, but carries a higher risk of serious complications, including occlusion and aneurysm formation affecting large vessels such as the aorta and peripheral arteries. The rarity and severity of arterial disease underscore its clinical significance when present.

In this report, we describe a 26‐year‐old male patient with recurrent mucocutaneous manifestations in whom the diagnosis of BD was established following presentation with severe left inguinal and proximal thigh pain, revealing extensive proximal arterial occlusion. This case is reported in accordance with the CAse Report (CARE) guidelines [7].

## 2. Case Presentation

A 26‐year‐old Palestinian male with a history of active smoking presented to the outpatient clinic with a several‐week history of left lower limb pain localized to the inguinal and proximal thigh regions. He also reported accompanying skin redness but denied swelling of the lower limb. The patient did not report any history of recent trauma, fever, or systemic symptoms, such as weight loss or night sweats. On physical examination, the patient exhibited localized tenderness, erythema, and increased warmth over the left inguinoscrotal region and proximal thigh.

The patient had a history of recurrent oral ulcers occurring approximately every 2 months, lasting for several days, and often resolving spontaneously. He also experienced occasional genital ulcers, which he described as painful and lasting approximately a week. Additionally, he reported episodic scrotal pain and redness, previously diagnosed as right epididymitis and orchitis. He denied any family history of autoimmune diseases or vasculitis and did not consume alcohol or drugs.

On physical examination, the patient exhibited tenderness at the left inguinoscrotal region and proximal thigh, with visible erythema and increased warmth over the affected area. The overlying skin showed no ulceration, rash, or bruising. There was no evidence of swelling in the limb, and the dorsalis pedis and posterior tibial pulses were bilaterally intact, with no signs of distal ischemia. Examination of the scrotum revealed mild erythema and tenderness without swelling or discharge. No active oral or genital ulcers were observed at the time of examination, and there were no cutaneous findings suggestive of pseudofolliculitis or erythema nodosum. Ophthalmologic screening did not reveal ocular redness or visual disturbance. Cardiovascular examination was unremarkable, with no murmurs or signs of heart failure, and peripheral pulses were symmetrical in other vascular territories. Neurological examination of the lower limbs was normal, with preserved motor strength, sensation, and reflexes.

Laboratory investigations revealed elevated inflammatory markers, with an erythrocyte sedimentation rate of 85 mm/h and a C‐reactive protein level of 51 mg/L. The white blood cell count was 18.9 × 10^9^/L, with neutrophil predominance. Hemoglobin level was 11.4 g/dL, while platelet count was within the normal range. Liver function tests were normal, with an alanine transaminase level of 29.2 U/L, and renal function was preserved, with a serum creatinine level of 1.0 mg/dL.

**FIGURE 1:**
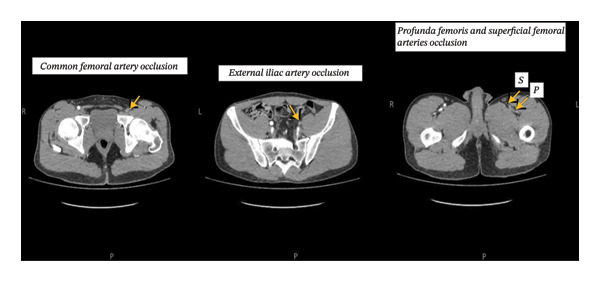
CT angiography of the left lower extremity showing complete occlusion of the common femoral artery, external iliac artery, profunda femoris, and superficial femoral arteries. No evidence of aneurysmal rupture or distal ischemia is noted, consistent with vascular Behçet’s disease.

Scrotal ultrasonography revealed mild right‐sided epididymitis with associated funiculitis, without signs of hydrocele or testicular torsion. Duplex ultrasonography of the left lower limb veins showed no evidence of deep venous thrombosis. Owing to persistent symptoms and concern for possible arterial involvement, computed tomography angiography was subsequently performed, which confirmed complete occlusion of the common femoral artery, external iliac artery, profunda femoris, and superficial femoral arteries (Figure 1).

**FIGURE 2:**
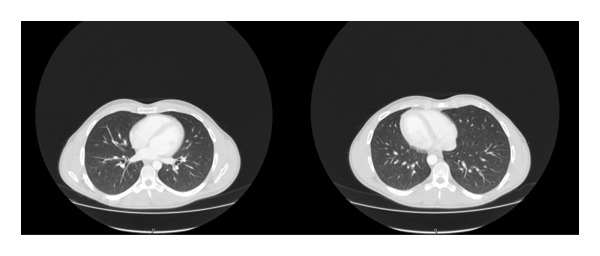
Chest CT imaging demonstrating no pulmonary vascular involvement. Representative axial contrast‐enhanced chest CT images (lung window) obtained to evaluate for pulmonary vascular complications associated with vascular Behçet’s disease. No evidence of pulmonary artery aneurysm, pulmonary artery thrombosis, or other thoracic vascular abnormalities is seen.

Given the known association between vascular BD and pulmonary artery involvement, additional evaluation for pulmonary vascular complications was performed. Chest imaging did not demonstrate evidence of pulmonary artery aneurysms, pulmonary artery thrombosis, or other thoracic vascular abnormalities (Figure 2). The absence of respiratory symptoms, hemoptysis, or signs of pulmonary hypertension further supported the lack of clinically significant pulmonary vascular involvement at the time of presentation.

Based on the patient’s clinical presentation, history of recurrent oral and genital ulcers suggestive of BD, and imaging findings, a diagnosis of vascular BD was established at the time of presentation. The patient was treated with high‐dose systemic corticosteroids, receiving methylprednisolone at a dose of 1 mg/kg/day. Immunosuppressive therapy with azathioprine was initiated at a dose of 2 mg/kg/day. In addition, low‐dose aspirin was prescribed.

The patient responded well to the pharmacotherapy regimen. Within 1 month of treatment, his symptoms significantly improved, with complete resolution of pain, erythema, and tenderness in the left lower limb. Repeat Doppler ultrasound and CT angiography demonstrated stabilization of the affected vascular segment, with no further dilation or evidence of thrombotic events. The patient was initially treated with high‐dose systemic corticosteroids in combination with azathioprine as induction therapy for active vascular inflammation. Following clinical improvement and radiological stabilization, corticosteroids were gradually tapered, and azathioprine was continued as maintenance immunosuppressive therapy to reduce the risk of disease relapse, with close clinical and radiological follow‐up.

## 3. Discussion

We report the case of a 26‐year‐old male with a history of recurrent oral and genital ulcerations and active smoking who presented with several weeks of left inguinal and proximal thigh pain associated with localized inflammatory signs. Imaging revealed extensive proximal arterial occlusion involving the common femoral, external iliac, profunda femoris, and superficial femoral arteries. Based on the combination of characteristic mucocutaneous manifestations and vascular imaging findings, a diagnosis of vascular BD was established at the time of presentation. The patient was treated with systemic corticosteroids and immunosuppressive therapy, with marked clinical improvement and radiological stabilization observed at 1‐month follow‐up. Although the diagnosis was established after the detection of arterial occlusion, recognition at the first vascular presentation allowed prompt initiation of immunosuppressive therapy, which may have contributed to disease stabilization and prevention of further complications. The following discussion focuses on aspects of BD directly relevant to the patient’s presentation and management, with particular emphasis on vascular involvement and therapeutic decision‐making.

BD is an inflammatory vasculopathy with multisystemic involvement. Diverse clinical manifestations typically accompany a relapsing–remitting clinical course [8]. Its prevalence is highest along the ancient Silk Road, with males often experiencing more severe disease [9], typically affecting individuals in their 20s or 30s [10].

Laboratory investigations in this case demonstrated markedly elevated inflammatory markers, including erythrocyte sedimentation rate and C‐reactive protein, along with leukocytosis. While no laboratory test is specific for BD, persistent systemic inflammation in a young patient with recurrent mucocutaneous manifestations should raise concern for potential vascular involvement. Recognition of sustained inflammatory activity may serve as an early warning sign prompting closer vascular surveillance and earlier intervention to prevent severe complications.

In our patient’s presentation, the history of recurrent oral ulcers and occasional genital ulcers provided important clinical context supporting the diagnosis of BD [11]. Vascular involvement occurs in up to approximately 40% of patients with BD, with venous manifestations being more frequent, while arterial involvement is less common but associated with a higher risk of severe complications, including occlusion and aneurysm formation [12].

These symptoms are critical in diagnosing BD, as they often precede other complications. While our patient did not report ocular symptoms, it is essential to recognize that ocular involvement is common in BD and can lead to severe visual impairment [13, 14]. The potential for associated neurological manifestations, such as cranial nerve palsies, further underscores the systemic nature of the disease [15]. This correlation highlights the multifaceted clinical features of BD and the necessity for comprehensive monitoring and management, especially considering the vascular complications evident in our patient’s case.

Vascular involvement represents a significant manifestation of BD, occurring in up to 40% of affected patients [16]. Venous lesions are observed more frequently than arterial involvement, and when arteries are affected, aneurysmal changes are reported more often than occlusive lesions [17]. The pattern and distribution of arterial disease vary among different ethnic populations. The data from Egypt indicate that the pulmonary artery and abdominal aorta are the most commonly involved sites, accounting for 44.4% and 14.8% of arterial aneurysms, respectively [18]. In contrast, studies from Turkey and Korea have identified the femoral artery and abdominal aorta as the predominant locations of arterial involvement [19, 20]. Overall, vascular complications are reported four to five times more frequently in males and tend to occur early in the course of the disease [21]. In the present case, computed tomography angiography demonstrated femoral artery involvement with occlusive changes and aneurysmal dilation, aligning with previous reports describing a predilection for lower extremity vascular disease in BD. Active smoking may have contributed to the severity of vascular inflammation observed in this patient.

According to the 2018 EULAR recommendations for the management of vascular BD, arterial involvement should be treated primarily with immunosuppressive therapy, including high‐dose systemic corticosteroids combined with an immunosuppressive agent, such as cyclophosphamide or azathioprine [22]. Antiplatelet therapy may be considered on an individual basis, particularly in the absence of contraindications, although its role remains adjunctive to immunosuppression [23]. In the present case, treatment consisted of systemic corticosteroids, azathioprine, and low‐dose aspirin, in accordance with these recommendations, with clinical and radiological stabilization observed during follow‐up.

Although arterial involvement in BD is uncommon and often considered a marker of severe disease, therapeutic decisions should be individualized according to clinical severity, extent of ischemia, and disease activity. Current EULAR recommendations advocate the use of high‐dose systemic corticosteroids combined with cyclophosphamide or biologic agents, particularly in cases complicated by arterial aneurysm formation, critical ischemia, or rapidly progressive vascular disease.

In the present case, despite extensive proximal arterial occlusion, the patient exhibited no clinical or radiological evidence of critical limb ischemia, aneurysmal rupture, distal hypoperfusion, or tissue loss. Peripheral pulses were preserved, and symptoms were limited to localized inflammatory pain without functional compromise. In this context, a decision was made to initiate high‐dose systemic corticosteroids in combination with azathioprine as an early immunosuppressive strategy, rather than immediate escalation to cyclophosphamide or antitumor necrosis factor therapy.

Additional factors influencing this therapeutic approach included the absence of life‐threatening vascular complications, the patient’s favorable initial clinical response, and limited local availability of biologic agents. The subsequent rapid clinical improvement and radiological stabilization support that, in carefully selected patients with non–life‐threatening arterial involvement, azathioprine may represent an effective immunosuppressive option when used in combination with systemic corticosteroids, with close monitoring for disease progression and readiness to escalate therapy if required.

For this patient with vascular BD, low‐dose aspirin was a critical component of the treatment plan, addressing the heightened risk of thromboembolic events due to arterial occlusions and vascular inflammation. Aspirin’s antiplatelet effect helped prevent further clot formation by inhibiting platelet aggregation, complementing the primary immunosuppressive therapy aimed at controlling inflammation. Its inclusion in the regimen contributed to the stabilization of vascular lesions and supported the patient’s overall positive clinical outcome, without introducing significant bleeding risks [24].

According to the 2018 EULAR recommendations, surgical or endovascular interventions in BD are primarily indicated for arterial aneurysms, particularly in cases with a high risk of rupture or other life‐threatening complications [25]. These procedures should ideally be undertaken after adequate control of vascular inflammation with immunosuppressive therapy, as active vasculitis is associated with an increased risk of postoperative complications. In contrast, arterial occlusive disease, as observed in the present case, is generally managed with medical therapy. In our patient, surgical or endovascular intervention was not pursued because there were no clinical or radiological signs of critical limb ischemia, such as claudication, rest pain, tissue loss, or hemodynamic compromise on Doppler ultrasonography or computed tomography angiography. Accordingly, a conservative medical approach with immunosuppressive therapy was favored, with subsequent clinical and radiological stabilization.

In conclusion, we report a case of extensive proximal arterial occlusion revealing vascular BD in a young male patient without clinical evidence of limb ischemia. The patient was successfully managed with immunosuppressive therapy alone, achieving clinical improvement and radiological stabilization without the need for surgical or endovascular intervention. This case highlights the importance of recognizing vascular BD at its initial vascular presentation and supports a conservative medical approach in carefully selected patients, with close follow‐up to monitor disease evolution.

## Author Contributions

Alhareth M. Amro, Leen M. Safadi, and Huda Barqawi: conceptualization, investigation, data curation, writing–original draft, and writing–review and editing. Ammar Shaheen and Saed I. Atawnah: supervision.

## Funding

The authors received no specific funding for this work.

## Ethics Statement

The authors have nothing to report.

## Consent

Written informed consent was obtained from the patient’s legal guardian for publication of this case report and any accompanying images. A copy of the written consent is available for review by the Editor‐in‐Chief of this journal.

## Conflicts of Interest

The authors declare no conflicts of interest.

## Data Availability

The data supporting the findings of this case report are available from the corresponding author upon reasonable request and with appropriate ethical approval. All data are anonymized to protect patient privacy and confidentiality, in accordance with journal data protection and privacy policies.
